# Complete Blood Count Reference Intervals for Healthy Han Chinese Adults

**DOI:** 10.1371/journal.pone.0119669

**Published:** 2015-03-13

**Authors:** Xinzhong Wu, Min Zhao, Baishen Pan, Jie Zhang, Mingting Peng, Lanlan Wang, Xiaoke Hao, Xianzhang Huang, Runqing Mu, Wei Guo, Rui Qiao, Wenxiang Chen, Hong Jiang, Yueyun Ma, Hong Shang

**Affiliations:** 1 Department of Laboratory Science, the Second Affiliated Hospital of Guangzhou University of Chinese Medicine, Guangdong, China; 2 Department of Clinical Laboratory Medicine, the First Affiliated Hospital of China Medical University, Shenyang, China; 3 Department of Clinical Laboratory Medicine, Zhongshan Hospital of Fudan University, Shanghai, China; 4 Department of Laboratory Medicine, Peking University Third Hospital, Beijing, China; 5 National Center for Clinical Laboratories, Beijing Hospital, Beijing, China; 6 Department of Laboratory medicine, West China Hospital, Sichuan University, Sichuan, China; 7 Department of Clinical Laboratory Medicine, Xijing Hospital, the Fourth Military Medical University, Xi'an, Shaanxi, China; University of Utah School of Medicine, UNITED STATES

## Abstract

**Background:**

Complete blood count (CBC) reference intervals are important to diagnose diseases, screen blood donors, and assess overall health. However, current reference intervals established by older instruments and technologies and those from American and European populations are not suitable for Chinese samples due to ethnic, dietary, and lifestyle differences. The aim of this multicenter collaborative study was to establish CBC reference intervals for healthy Han Chinese adults.

**Methods:**

A total of 4,642 healthy individuals (2,136 males and 2,506 females) were recruited from six clinical centers in China (Shenyang, Beijing, Shanghai, Guangzhou, Chengdu, and Xi’an). Blood samples collected in K2EDTA anticoagulant tubes were analyzed. Analysis of variance was performed to determine differences in consensus intervals according to the use of data from the combined sample and selected samples.

**Results:**

Median and mean platelet counts from the Chengdu center were significantly lower than those from other centers. Red blood cell count (RBC), hemoglobin (HGB), and hematocrit (HCT) values were higher in males than in females at all ages. Other CBC parameters showed no significant instrument-, region-, age-, or sex-dependent difference. Thalassemia carriers were found to affect the lower or upper limit of different RBC profiles.

**Conclusion:**

We were able to establish consensus intervals for CBC parameters in healthy Han Chinese adults. RBC, HGB, and HCT intervals were established for each sex. The reference interval for platelets for the Chengdu center should be established independently.

## INTRODUCTION

Complete blood count (CBC) reference intervals are essential for effectively diagnosing diseases, screening blood donors, and assessing overall health. However, critical accuracy gaps exist in the reference intervals that are currently used in China. Most available reference intervals for laboratory tests are outdated or adopted from the manufacturers of the diagnostic tests. Over the past 30 years, the nutritional status and lifestyles of Chinese people have undergone dramatic changes. Thus, the reference intervals established using older instruments and technologies might no longer be relevant. In addition, manufacturers’ reference intervals, which were predominantly established for North American and European populations, may not account for variations due to race, diet, and lifestyle. Therefore, there is an urgent need to establish accurate CBC reference intervals for Chinese people.

The use of different methods, instruments, and populations can lead to significant differences in CBC parameters, such as hemoglobin (HGB), hematocrit (HCT), red blood cell count (RBC), and white blood cell count (WBC) [1, 2]. Advances in standardizing methods and laboratory techniques have minimized the effects of site-to-site differences in many analytical methods [3]. Population is one of the key remaining variables that can influence a reference interval. The ethnic Han population is the predominant population in Mainland China. Therefore, a common reference interval for CBC parameters in Han Chinese adults should be established via multicenter collaborative studies using uniform standards.

## MATERIALS AND METHODS

### Ethical considerations

The Ministry of Health and Clinical Laboratory Branch of the Chinese Medical Association supported a multicenter research study to establish reference intervals for major clinical laboratory items in the Han Chinese population. Six centers were selected to participate in this research: the Second Affiliated Hospital of Guangzhou University of Chinese Medicine, First Affiliated Hospital of China Medical University, Zhongshan Hospital of Fudan University, Peking University Third Hospital, West China Hospital of Sichuan University, and Xijing Hospital of the Fourth Military Medical University. Each hospital was responsible for recruiting healthy local candidates, collecting samples, and performing tests. The National Center of Clinical Laboratories (NCCL) was responsible for project quality assurance.

This study was approved by the Research and Ethics committees of the six participating hospitals. Each participant signed consent forms before undertaking any study-related activities.

### Subject enrollment

Healthy ethnic Han male and female candidates between 20 and 79 years of age were screened and enrolled across the six clinical research centers, representing the six official geographical regions in China (Shenyang, Beijing, Shanghai, Guangzhou, Chengdu, and Xi’an). At enrollment, demographic information and medical history were collected, a physical examination was performed, and blood and urine samples were obtained. After prescreening, potentially eligible subjects with positive laboratory screening results were excluded based on the following criteria:

1. Diagnosed disease, including acute or chronic infection, atherosclerosis, empyrosis, renal failure, hyper- or hypothyroidism, diabetes, tumor, or hepatic, heart, allergic, hematopoietic, or respiratory disease;
2. Surgery within 6 months after enrollment;
3. Blood donation or transfusion within 4 months of enrollment;
4. Body mass index exceeding 28 kg/m^2^;
5. Pregnancy;
6. Hypertension;
7. Use of alcohol, tobacco, or oral contraceptives;
8. Positivity for hepatitis B surface antigen (HBsAg), hepatitis C antibody (anti-HCV), or human immunodeficiency virus antibody (anti-HIV);
9. Abnormal urinalysis results, including the presence of erythrocytes, granulocytes, glucose, protein, or nitrite;
10. Plasma fasting blood glucose (FBG) level exceeding 7.0 mmol/L;
11. Serum iron level lower than 6 μmol/L;
12. HGB concentration less than 90 g/L;
13. WBC less than 3.0×10^9^/L or more than 12.5×10^9^/L; and
14. Thalassemia trait (TT) or carrier (Guangzhou and Chengdu centers only).

From September 2010 to January 2011, 18,000 volunteers were recruited. Of them, 7,612 volunteers (42%) were enrolled, but 4,642 volunteers (26%; 2,136 males and 2,506 females) were included in the final analysis to create the CBC reference intervals. A large number of volunteers were excluded because of abnormal physical examination results or positive laboratory screening results. Reference subjects were divided into six age groups: 20–29, 30–39, 40–49, 50–59, 60–69, and 70–79 years (Table 1). The ratio of subjects from the city and surrounding rural area was 7:3.

**Table 1:** Population Demographics for Establishing the Reference Range by Center.

ClinicalCenter	Number	Sex			Age (years)						
		M	F		Mean	20–29	30–39	40–49	50–59	60–69	70–79
Shenyang	861	368	493		43.9	186	145	203	188	97	42
Beijing	760	340	420		43.7	177	155	136	148	83	61
Shanghai	736	292	444		44.0	156	146	129	188	101	16
Guangzhou	945	446	499		47.8	179	162	159	162	149	134
Chengdu	644	331	313		41.4	157	150	157	114	53	13
Xi’an	696	359	337		38.4	228	162	128	121	44	13
Total	4 642	2 136	2 506		43.5	1 083	920	912	921	527	279

### Instruments and measurement parameters

All six centers measured CBC parameters with XE-2100 (Sysmex Corp., Kobe, Japan) and BC-5800 (Mindray Medical Electronics Co., Shenzhen, China) hematology analyzers. Measured CBC parameters included RBC, HGB, HCT, mean corpuscular volume (MCV), mean corpuscular hemoglobin (MCH), MCH concentration (MCHC), platelet count (PLT), WBC, and WBC differentials, including percentages and absolute counts of neutrophils (NEUT% and NEUT), lymphocytes (LYM% and LYM), monocytes (MONO% and MONO), basophils (BASO% and BASO), and eosinophils (EO% and EO).

### Sample collection and analysis

Each participant fasted from food and water for at least 8 hours but no more than 14 hours. Blood from each participant was drawn from the cubital vein into appropriate blood collection tubes using vacuum tube needles (Becton Dickinson Medical Devices Co. Ltd., Franklin Lakes, USA). K2EDTA tubes were used for CBC and DNA analyses. Fluoride oxalate tubes were used for FBG analysis. Plain tubes were used for estimating serum iron, anti-HCV, anti-HIV, and HBsAg levels. Samples collected in fluoride oxalate or plain tubes were separated by centrifugation for 10 minutes at 3,000 rpm. All enrolled subjects provided urine samples for urinalysis. Samples were transported and tested within 4 hours after collection.

Among the six centers, the prevalence of thalassemia was high in Guangzhou and Chengdu [4]. At these centers, an MCV value of less than 82 femtoliters (fL) promoted DNA analysis to screen for thalassemia carriers. Gap-polymerase chain reaction (PCR) was used to detect three routine gene deletion types in the α-thalassemia gene (-α^3.7^, -α^4.2^, and —^SEA^). Seventeen common mutants of β-thalassemia in the Chinese population were detected by PCR reverse dot-blot hybridization (PCR-RDH; YANENG Bioscience Co. Ltd., Shenzhen, China) [5,6].

### Quality assurance

According to Clinical and Laboratory Standards Institute (CLSI) guideline C28-A3 [3], the compatibility of different measurement systems must be established when a multicenter reference interval study is performed. Before collecting samples for final analysis, the performance of the automated hematology analyzers was evaluated. After calibration by a field service representative, the imprecision, carry over, and linearity of the analyzers were evaluated according to the International Council for Standardization in Haematology (ICSH) guidelines [7]. Accuracy tests were performed monthly throughout the entire study, in accordance with a standard protocol. Fresh K2EDTA anticoagulant specimens were prepared by the NCCL and tested within 8 hours after sample collection. Test results were compared with reference values defined by the NCCL. The acceptable range of bias for WBC, RBC, HCT, HGB, and PLT was half of the allowable error described by the Clinical Laboratory Improvement Amendments of 1988 (CLIA 88) [8].

To ensure standardization, all laboratory staff received equipment training and were required to pass an independent quality review before enrolling volunteers. The procedure and timing of blood sample collection were standardized to minimize the preanalytical variables. Commercial control samples at three different levels (high, medium, and low) were measured before and after every analytical run.

### Statistical analysis

Data were analyzed with the Statistical Package for Social Sciences (SPSS version 17.0) and Microsoft Excel 2003. Outliers were excluded by the one-third rule for the D/R ratio, where D is the absolute difference between an extreme observation (large or small) and the next largest (or smallest) observation, and R is the range of all observations, including extremes [3]. Multiple linear regression was performed to explore impact factors. Step-wise regression was used to screen variables. Nested analysis of variance (ANOVA) was performed to analyze data by instrument, clinical center, age, and sex. For parameters with a skewed distribution, all ANOVA tests were performed after a log transformation, and the geometric means were compared instead of the arithmetic means.

If the overall F-test from an ANOVA on mean values was statistically significant (P < 0.05), then a step-wise procedure was used to determine which intervals were close enough to combine into a “consensus interval” [9]. First, we compared the intervals between the two most similar sites based on the P-values from the overall ANOVA adjusted for multiple comparisons by the Tukey method. Data from the two sites were combined if: 1) the difference was not statistically significant, 2) the difference between the means was significant, but was less than 25% of the width of the 95% reference interval estimated from the combined sample, and the ratio of standard deviations (SDs) was less than 1.5. The combined data were compared to each of the remaining sites as described above, using a new ANOVA. These steps were repeated until all of the sites were combined into or excluded from the consensus interval. Finally, the consensus intervals for men and women were compared and combined, if appropriate, in the same manner. Nonparametric methods were used to establish the CBC consensus intervals, which were calculated using CBC values between the 2.5^th^ and 97.5^th^ percentiles that included 95% of the reference sample group data.

## Results

### Region-related findings

**Fig 1:**
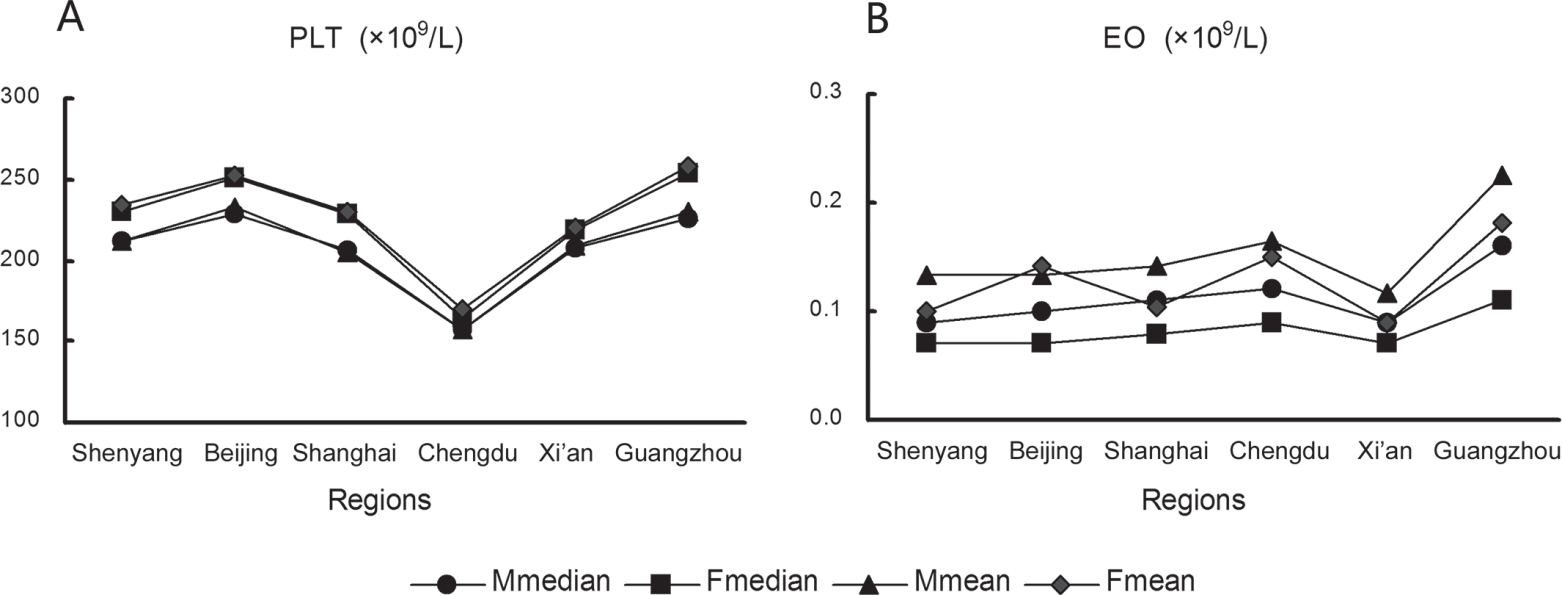
Median and mean platelet counts (PLT, A) and eosinophil (EO, B) values for different regions and sexes.

Median and mean values of the PLT parameter in samples from the Chengdu center were significantly lower than values from other centers (Fig. 1A; *P* < 0.05). The difference between means was greater than 25% of the width of the 95% reference interval estimated from the combined sample data; therefore, data from the Chengdu center were excluded from the consensus interval for PLT. Median and mean EO values were significantly greater in samples from the Guangzhou center than in those from other centers (Fig. 1B; *P* < 0.05). However, the difference between means was minor, and EO data from all centers could be combined to calculate the consensus interval.

### Instrument-related findings

**Fig 2:**
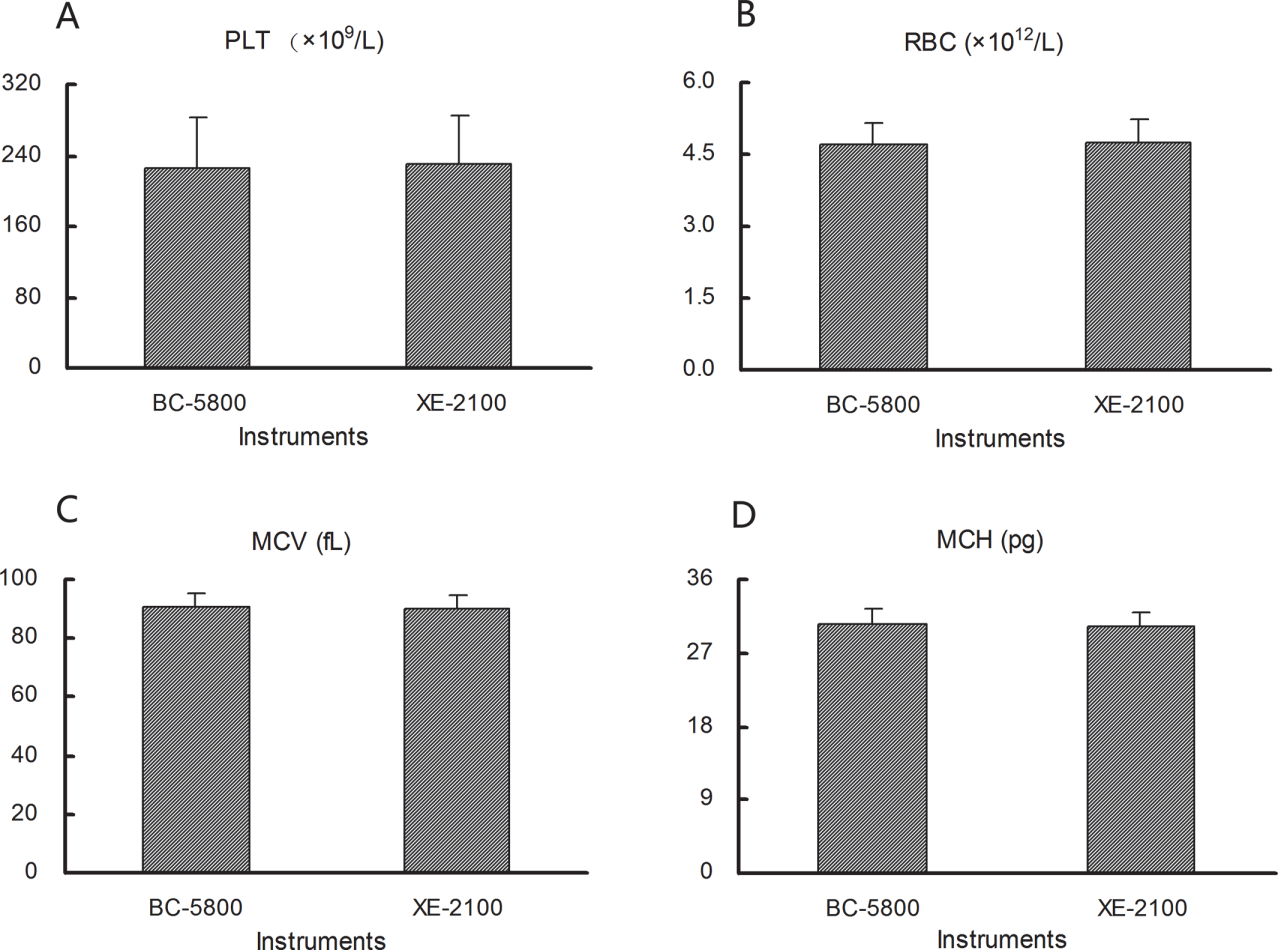
Mean platelet count (PLT, A), red blood cell count (RBC, B), mean corpuscular volume (MCV, C), and mean corpuscular hemoglobin (MCH, D) value according to the use of different instruments.

No statistically significant differences in the RBC, MCV, MCH, and PLT parameters were observed between the Mindray BC-5800 and SYSMEX XE-5000 analyzers (Fig. 2A-2D; *P* > 0.05). Similar results were obtained for HGB, HCT, MCHC, WBC, and WBC differentials between the two instruments (data not shown; *P* > 0.05). Thus, the data obtained for each parameter from each instrument could be pooled to determine the consensus intervals.

### Age- and sex-related findings

**Fig 3:**
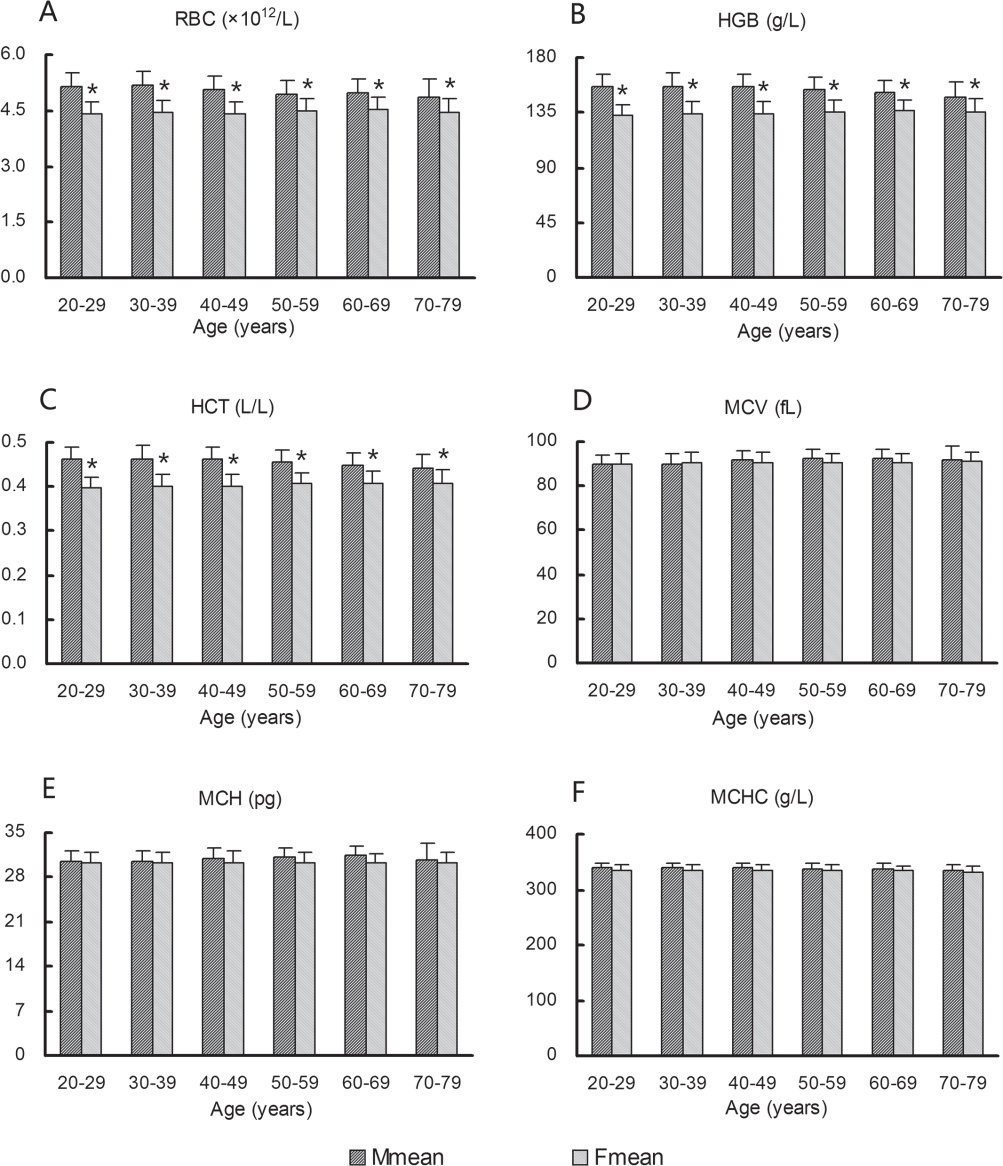
Variations in red blood cell (RBC) profile according to age and sex. (A) RBC, (B) hemoglobin (HGB), (C) hematocrit (HCT), (D) mean corpuscular volume (MCV), (E) mean corpuscular hemoglobin (MCH), (F) MCH concentration (MCHC). *Females show significantly lower values (*P* < 0.05) than males in the same age group.

Fig. 3A-3C display mean values of RBC, HGB, and HCT according to age and sex. A significant difference between sexes (*P* < 0.05) and a minor difference among age groups were observed. Thus, RBC, HGB, and HCT data from different age groups were pooled, but the sex distinction was preserved when establishing reference intervals. RBC, HGB, and HCT values tended to decrease with age in males and were obviously reduced in the 70–79 age group, and these values tended to increase slightly with age in females and were lower than values in males in all age groups. Mean values of MCV, MCH, and MCHC did not differ according to age or sex in the combined analysis of all data (Fig. 3D-3F).

**Fig 4:**
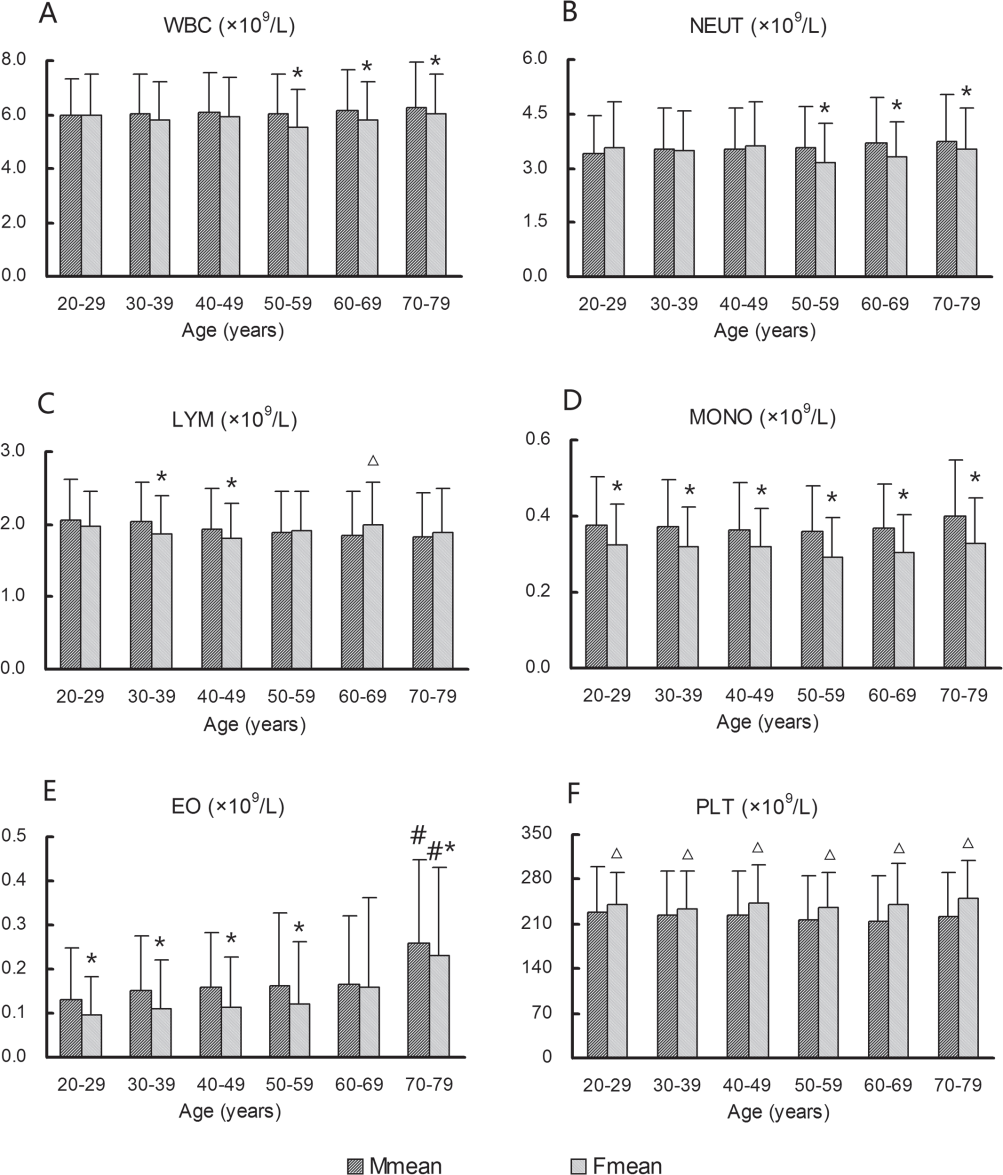
Variations in white blood cell (WBC) profile and platelet count (PLT) according to age and sex. (A) WBC, (B) neutrophils (NEUT), (C) lymphocytes (LYM), (D) monocytes (MONO), (E) eosinophils (EO), (F) PLT. *Females show significantly lower values (*P* < 0.05) than males in the same age group. △Females show significantly higher values (*P* < 0.05) than males in the same age group. #EO values are significantly higher (*P* < 0.05) in the 70–79-year age group than in other age groups in males and females, respectively.

Mean values for the WBC and NEUT parameters were slightly increased in males over 60 years, and were higher in males than in females over the age of 50 years (Fig. 4A and B, *P* < 0.05). Conversely, LYM values showed a decreasing trend with age in males (Fig. 4C). EO values in males and females tended to increase with age, with an apparent increase in the 70–79-year age group (*P* < 0.05). EO and MONO counts appeared to be consistently lower in females than in males across all age groups (Fig. 4D and 4E, *P* < 0.05). In contrast, mean platelet counts were significantly higher in females than in males in all age groups (Fig. 4F, *P* < 0.05). However, these differences were not sufficiently significant to prompt exclusion from study consensus intervals. Apart from RBC, HGB, and HCT, data for other CBC parameters could be combined to calculate consensus intervals, regardless of age or sex.

### Relationship between TT and reference intervals for RBC parameters

**Fig 5:**
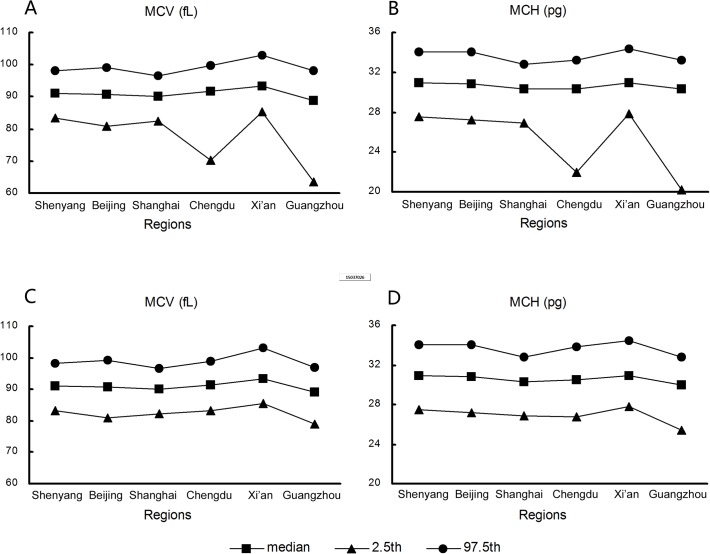
Mean corpuscular volume (MCV) and mean corpuscular hemoglobin (MCH) reference intervals for samples with and without thalassemia trait (TT). Percentiles for (A) MCV with TT, (B) MCH with TT, (C) MCV without TT, (D) MCH without TT according to regions.

At the Guangzhou and Chengdu centers, TT carriers were identified based on MCV values and thalassemia gene DNA analysis. TT carriers accounted for 10.5% (170/1,622) and 4.3% (39/912) of subjects from these centers, respectively. Inclusion of TT carriers from these centers in the final analysis clearly affected the lower limits of the MCV, HGB, and MCH parameters and the upper limit of the RBC parameter (Table 2, Fig. 5A and 5B), but had little or no impact on the HCT and MCHC intervals. After the exclusion of TT carriers, differences in RBC, HGB, MCV, and MCH percentiles among the six centers were minor, and all data could be combined to determine the consensus intervals for each parameter (Fig. 5C and 5D). TT carriers had an obvious effect on the 2.5^th^ percentiles, but negligible impacts on the median and 97.5^th^ percentiles of the MCV and MCH intervals.

**Table 2:** Reference Intervals for RBC Profiles with and without TT Carriers.

Parameters	Sex	Chengdu			Guangzhou	
		With TT	Without TT		With TT	Without TT
RBC (×10^12^/L)	M	4.29–6.20	4.29–5.66		4.34–6.88	4.31–5.94
RBC (×10^12^/L)	F	3.83–5.02	3.83–4.95		3.92–5.83	3.91–5.24
HGB (g/L)	M	129–171	133–171		125–176	133–179
HGB (g/L)	F	109–147	110–147		111–153	115–153
HCT (L/L)	M	0.40–0.51	0.41–0.51		0.39–0.51	0.40–0.51
HCT (L/L)	F	0.35–0.45	0.35–0.45		0.34–0.45	0.35–0.45
MCV (fL)	M/F	74.0–98.9	83.6–99.0		64.0–96.6	78.6–96.8
MCH (pg)	M/F	22.7–32.7	26.5–32.7		20.3–32.8	25.4–32.9
MCHC (g/L)	M/F	308–344	310–345		311–356	317–356

### Establishing final consensus intervals

Apart from RBC, HGB, and HCT, the consensus CBC parameters did not differ by sex. Consensus intervals obtained by using the 2.5^th^ and 97.5^th^ percentiles as the lower and upper limits, respectively, are shown in Table 3. According to the CLSI C28-A3 guideline [3], CBC consensus reference intervals may be required to validate the transference. For the previously described screening standards, data from at least 20 new male and female subjects were collected from each of the six centers to verify the consensus intervals. A total of 309 individuals (149 males and 160 females) were selected to verify the consensus intervals. The qualified rate of validation for all of the analytes was not less than 95%. The analytes that most commonly exceeded the limits of the consensus intervals were HCT and MCHC, which were the two calculated parameters. Other previously published consensus intervals are shown for comparison in Table 3.

**Table 3:** Consensus intervals, validation rates, and comparative interval.

Parameter	Sex	Consensus interval	Qualified rate of validation	Current interval used in China	American	Malaysian	African
RBC (×10^12^/L)	M	4.28–5.81	97.3%	4.09–5.74	4.5–5.9	4.18–6.06	4.0–6.4
RBC (×10^12^/L)	F	3.81–5.13	96.3%	3.68–5.13	4.0–5.2	3.52–5.16	3.8–5.6
HGB (g/L)	M	133–175	99.3%	131–172	135–175	120–165	122–177
HGB (g/L)	F	115–152	96.9%	113–151	120–160	98–138	95–158
HCT (L/L)	M	0.40–0.51	95.3%	0.380–0.508	0.41–0.53	0.375–0.498	0.35–0.51
HCT (L/L)	F	0.35–0.46	96.3%	0.335–0.450	0.36–0.46	0.318–0.424	0.29–0.45
MCV (fL)	M/F	82.3–99.2	98.1%	M:83.9–99.1/F:82.6–99.1	80–100	M:78.9–95.7/F:77.5–94.5	68–98
MCH (pg)	M/F	27.0–33.7	98.4%	M:27.8–33.8/F:26.9–33.3	-	M:25.4–31.1/F:24.8–31.2	-
MCHC (g/L)	M/F	316–354	95.1%	M:320–355/F:322–362	-	M:30.6–34.8/F:29.4–64.4	-
WBC (×10^9^/L)	M/F	3.64–9.39	98.7%	M:3.97–9.15/F:3.69–9.16	4.5–11.0	M:3.8–9.7/F:3.4–10.1	3.1–9.1
NEUT (%)	M/F	41.5–73.8	99.0%	50–70	40–70	M:42.8–69.2/F:43.2–70.6	25–66
LYM (%)	M/F	18.6–48.7	98.7%	20–40	22–44	M:18.5–47.7/F:19.2–47.5	23–59
MONO (%)	M/F	3.2–9.5	97.1%	3–10	4–11	-	4.5–13.1
EO (%)	M/F	0.4–8.1	97.4%	0.5–5	0–8	-	0.8–21.8
BASO (%)	M/F	0.1–1.1	99.7%	0–1	0–3	-	0.4–2.5
NEUT (×10^9^/L)	M/F	1.80–6.30	99.0%	2.0–7.0	1.8–7.7	M:1.58–5.94/F:1.55–6.07	1.0–5.3
LYM (×10^9^/L)	M/F	1.06–3.20	98.4%	0.8–4.0	1.0–4.8	M:1.14–3.22/F:1.05–3.29	1.2–3.7
MONO (×10^9^/L)	M/F	0.16–0.62	98.1%	0.12–1.00	0–0.8	M:0.15–0.67/F:0.1–0.74	0.20–0.78
EO (×10^9^/L)	M/F	0.02–0.52	98.1%	0.02–0.50	0–0.45	M:0.08–0.28/F:0.03–0.27	0.04–1.53
BASO (×10^9^/L)	M/F	0.00–0.06	99.4%	0–1	0–0.2	M:0.01–0.05/F:0.01–0.05	0.01–0.15
PLT (×10^9^/L)*	M/F	127–350	97.4%	M:85–303/F:101–320	150–350	M:167–376/F:158–410	126–438
PLT (×10^9^/L)^#^	M/F	67–287	100%				

*: Five centers after exclusion of Chengdu,

^#^: only Chengdu center.

## Discussion

Reference methods for measuring the RBC, WBC, HGB, HCT and PLT parameters have been previously published by the ICSH [10–13]. The published values are comparable regardless of analyzer, reagent, or analytical principle. Consequently, the values from different laboratories or instruments are considered interchangeable, and it is possible to combine data from multiple centers to establish consensus reference intervals. Once the consensus reference intervals are established, receiving laboratories must only validate them by examining a small number (e.g., 20) of reference individuals from their subject populations [3].

There is relatively little published data available describing the CBC reference intervals for people in China. Following the CLSI guidelines and standardization protocol, we established consensus reference intervals for the ethnic Han population, which represents 98% of the population in China. To ensure the accuracy and validity of the results, quality assurance steps were taken in the preanalytical and analytical stages. We established CBC consensus intervals using statistical calculations and verified these intervals with a smaller sample of reference subjects.

CBC values are influenced by several factors, including race, age, sex, altitude, and smoking [14–16]. In this study, the influences of instrument type, age, sex, and region on CBC reference intervals were analyzed. Of the CBC parameters, only PLT showed significant regional variability. PLT values from male and female subjects in Chengdu were significantly lower than values from subjects at other sites and, therefore, were not included in the consensus intervals. The reason for the lower PLT values remains unclear, but could be partially attributable to the humid climate and lack of sunshine in the Sichuan basin. Significant sex-based differences in platelet counts were also observed in African studies [17,18]. The reasons for these differences are still unclear. The lower limit for the PLT parameter in our consensus intervals is slightly lower than what has been previously established for Americans [19] and Malaysians [20], but is higher than the current interval used in China [21].

The WBC and NEUT values increased slightly with age, which might be related to chronic infection in elderly people that were not excluded according to our criteria. Differences in EO values according to region, sex, and age group were identified, and may be attributable to the presence of allergic and parasitic diseases in the apparently healthy elderly subjects. For example, mites living in tropical and humid conditions [22] or pathogenic protozoa consumed in contaminated water and foods in Guangdong Province [23, 24] may have caused such diseases. The apparent increase in EO values in males and females aged 70–79 years is consistent with data from the Guangzhou center, from which almost 50% subjects were aged more than 70 years. We observed no significant sex-based difference in WBC values across our study populations. This finding was similar to data reported in the 2^nd^ National Guide to Clinical Laboratory Procedures, but different from those reported in the 3^rd^ National Guide [21, 25]. Compared with other published data, the reference intervals for WBC and NEUT were similar to intervals for Malaysians, lower than those for Americans, and higher than those for Africans [9].

The lower limits of the RBC, HGB, and HCT parameters decreased slightly with age in male subjects. This decrease could be due to the gradual loss of androgens, which stimulate increased production of erythrocytes [26, 27]. In contrast, RBC, HGB, and HCT values increased slightly with age in females, consistent with the lower levels of these parameters before menopause and higher levels after menopause [28]. The average RBC, HGB, and HCT values in males decreased at 70–79 years of age, which could contribute to nutritional deficiency, occult malignancy, and chronic anemia in the aging population. The same results were observed in North American populations [15]. In addition, the RBC, HGB, and HCT values were higher in males than in females at all ages due to chronic menstrual blood loss in females and a higher androgen level in males.

We identified regional variations in the MCV and MCH parameters that could be related to the high prevalence of TT in Guangzhou and Chengdu (Southern China). The prevalence of TT carriers we observed in Guangzhou (10.5%) and Chengdu (4.3%) were similar to previously published data [29, 30]. When TT carriers were excluded from analysis, the MCV and MCH parameters were not significantly different among the six centers. However, because thalassemia gene DNA analysis was only performed on enrolled individuals whose MCV was 82 fL or less, and as only 20 of the most typical DNA deletions or mutations were detected, there could be a small population with TT that were not excluded in our study. Nevertheless, when TT carriers with microcytic characteristics were excluded, the influence of TT carriers with MCV greater than 82 fL on the RBC reference intervals was negligible. Furthermore, the CLSI guidelines do not provide clear protocols for how to proceed when apparently healthy subjects have asymptomatic illnesses that impact the reference intervals. Our results suggest that in regions with a high prevalence of thalassemia, such as the Mediterranean, the Middle East, Africa, and Southeast Asia [9, 20, 31, 32], it is critical to exclude TT carriers from healthy candidates to establish RBC reference intervals. Different methods, such as hemoglobin electrophoresis, RBC indices, blood film, HPLC analysis, or DNA analysis, can be used to screen reference individuals [33–35].

The lower limit of MCV in our consensus intervals was slightly higher than those in America [19] and Malaysia [20], and substantially higher than that in Africa [9]. The MCV consensus interval was similar to American populations with a low prevalence of thalassemia, and similar to the lower limit of the Malaysian population after excluding TT carriers with the Biorad Variant II hemoglobin analyzer [20]. Without exclusion of TT carriers, the decreased lower limit of MCV is more obvious in CBC reference intervals of the African population [9]. The lower limit of the HGB reference interval has substantially increased in the Chinese population over the past 30 years compared to previous studies [25].

With a sufficient sample size, standard statistical analyses can detect significant differences, but that does not necessarily mean that the differences are clinically significant for the subjects. In our study, most parameters (e.g., MCV, RBC, MCH, WBC, NEUT, and PLT) had some variations across instruments, regions, age, or sex. However, the variations were not significant, and all of the data could be combined to calculate the consensus intervals. Reference intervals derived from different regions, instruments, and ages might appear to be more conservative in clinical application. The present study is the largest published study to define CBC reference intervals in China. As China is a vast country with a large population, it is reasonable to assume that CBC parameters could vary regionally and in a population-dependent manner. Our multicenter study addresses this possible variation and establishes consensus CBC reference intervals (except for PLT) for healthy Han Chinese adults. The established laboratory intervals will aid in clinical diagnosis and prognosis analyses.
